# TEACHING AND LEARNING IN TURBULENT TIMES

**DOI:** 10.1093/geroni/igae098.0304

**Published:** 2024-12-31

**Authors:** Lyn Holley

**Affiliations:** University of Nebraska at Omaha, Omaha, Nebraska, United States

## Abstract

Teachers now face circumstances in which everything, everywhere, seems to be changing all at once. The ongoing techno-communications revolution is continuous and relentless. Many agree that its impacts will equal those of the Industrial Revolution. With particular emphasis on Lifelong Learning and Intergenerational engagement, the recipient of the 2024 AGHE Distinguished Faculty Award will lead discussion of ongoing changes in traditional education, and then will describe some strategies for engaging existing resources and developing approaches to pedagogy and andragogy that are useful for coping with the continuous change of the current foundations of formal education. Finally, learning opportunities provided by GSA and AGHE annual conferences and by membership in GSA and AGHE will be discussed.

